# Grit and Work Engagement: A Cross-Sectional Study

**DOI:** 10.1371/journal.pone.0137501

**Published:** 2015-09-03

**Authors:** Yuhei Suzuki, Dai Tamesue, Kentaro Asahi, Yoshiki Ishikawa

**Affiliations:** 1 Campus for H, Shibuya-ku, Tokyo, Japan; 2 Research Center for Advanced Science and Technology, The University of Tokyo, Meguro-ku, Tokyo, Japan; 3 Beach Life JAPAN, Chuo-ku, Tokyo, Japan; 4 Department of Health and Social Behavior, School of Public Health, The University of Tokyo, Bunkyo-ku, Tokyo, Japan; Hamamatsu University School of Medicine, JAPAN

## Abstract

Grit, defined as perseverance of effort and consistency of interest, has attracted attention as a predictor of success in various fields beyond IQ and the Big Five personality dimension of Conscientiousness. The purpose of the current study was to examine previously uninvestigated questions regarding grit using a cross-sectional design among a large number of working adults in Japan. First, we tested geographical generalizability of associations between grit and orientations towards happiness by comparing previous studies in the U.S. and the current study in Japan. It was confirmed that orientation towards meaning rather than orientation towards engagement had a stronger positive correlation with grit in our sample of Japanese people. This result is inconsistent with previous studies in the U.S. Furthermore, the Big Five dimension of Openness to Experience was newly confirmed as having a positive association with grit. Second, we examined the association between grit and work engagement, which is considered as an outcome indicator for work performance. In this analysis, grit was a strong predictor for work performance as well as academic performance.

## Introduction

The question “what’s the key factor for success?” has long been an important theme in the field of occupational psychology. As many studies have shown, intelligence or general mental ability is one of the key factors (e.g., [1]). Furthermore, IQ is a well established index that predicts work outcomes more reliably and precisely than any other stable individual personality trait (e.g., [2]). However, an unanswered question remains: “Why are there people who achieve success and others who do not, although they have the same level of intelligence?” Far less is known about individual differences other than IQ that predict success. Previous studies have established Conscientiousness, one of the Big Five personality traits, as another general predictor for high performance across various fields (e.g., [3]), but there are still dimensions or traits that have not been explored yet.

A newly defined personality trait called “grit” has been attracting the interest of scholars in recent years. Duckworth *et al*. [4] defined grit as a personality trait of perseverance of effort and consistency of interest for long-term goals, and suggested grit as a valid predictor of long-term success shared by the most prominent leaders in every field. Grit did not show positive correlations with IQ but correlated highly with the Big Five dimension of Conscientiousness, and demonstrated incremental predictive validity of success measures over and beyond IQ and Conscientiousness.

In addition, Von Culin *et al*. [5] suggested that individual differences in grit may derive in part from differences in orientations towards happiness. In short, grit demonstrated medium-sized associations with an orientation towards engagement, small-to-medium associations with an orientation towards meaning, and small-to-medium (inverse) associations with an orientation towards pleasure. Based on their study, we suggest that those who feel happiness when engaging in activities are more likely to achieve success.

However, since grit is still a newly developed measure, several questions need to be investigated before the concept of grit can be generalized. First, at the population level, it is unclear whether Duckworth *et al*.*’s* [4] hypothesis and their results on grit are replicable in geographical and ethnical regions other than the U.S. Especially in Japan, where Kumano [6] suggested that people have different orientations towards happiness and different factors influence life satisfaction, it is possible that the predictability of grit for success and its correlation with orientations towards happiness may differ. Second, for the outcome level, it is unclear whether grit is a valid predictor of work performance across all occupations. In their previous study, Duckworth *et al*. [4] mainly focused on the association between grit and academic achievements such as educational attainment and grade point average (GPA). As for professional success outcomes, they analyzed associations between grit and retention rate in military schools or number of lifetime career changes. Therefore, we do not know to what extent grit affects actual work performance in a business environment.

Our current study pursued two research questions by replicating Duckworth *et al*.*’s* [4] studies about grit in Japan and answering novel questions about the associations between grit and work performance. First, we measured associations between grit and orientations towards happiness. Our first hypothesis was that engagement and meaning would show positive correlations with grit, while pleasure would show negative correlations, which is in accordance with Von Culin *et al*.*’s* study [5]. In addition to orientations towards happiness, we also tested the associations between grit, the Big Five personality trait of Conscientiousness, and self-control. As Duckworth *et al*. [4] discussed, grit overlaps with the achievement aspect of Conscientiousness, by which people make efforts to achieve goals, and with the dependability aspect of Self-Control, by which people effectively control their tempers. Therefore, we expected that both Conscientiousness and self-control would exhibit some level of association with grit.

Second, we tested associations between grit and a major work performance indicator, the Utrecht Work Engagement Scale (UWES). Bakker [7] defined work engagement as a positive, fulfilling work-related state of mind that is characterized by vigor, dedication, and absorption, and that is used to predict high work performance in organizations. Because vigor—namely, the willingness to invest effort in one’s work—overlaps with the grit subscale perseverance of effort, our second hypothesis was that grit would be positively and strongly associated with work engagement. The current study examined whether grit is important to performing well in a business environment, where working people need to socially interact with others rather than studying hard by themselves to achieve a high GPA or education attainment, which Duckworth *et al*. [4] used as outcomes. In addition to grit, we also tested associations between work engagement and orientations to happiness, Big Five traits, and self-control. Because dedication relates to a worker’s sense of significance, enthusiasm, inspiration, pride, and challenge, the orientation towards meaning—one of the orientations towards happiness—should be positively associated with work engagement. In addition, as absorption is characterized by being fully concentrated and happily engrossed in one’s work, the orientation towards engagement should also be positively associated with work engagement. As mentioned for the first research question, Conscientiousness and self-control share similar aspects with grit; we therefore expected that they would also have positive associations with work engagement. However, grit differs from Conscientiousness in its emphasis on long-term stamina rather than short-term intensity, and it differs from self-control in its specification of consistent goals and interests. Therefore, we hypothesized that grit would have an incremental predictive validity for work engagement over and above those of Conscientiousness and self-control.

## Method

### Study Design

For the question 1, we examined the associations between grit and three different happiness orientations, Big Five personality traits, self-control level, and various demographic variables in Japanese adults using a cross-sectional design. We specifically examined to which extent those personality traits and demographic variables explain variance in grit and its two facets: perseverance and consistency of interest. For the question 2, we examined the associations between the explanatory variables of grit and other personality and demographic scales and the outcome variable of UWES score in Japanese adults using a cross-sectional design.

### Participants

Participants in the current study consisted of 1,134 adults (50.79% female, mean age 43.13 years) who voluntarily completed self-report questionnaires that we had developed. Those who answered the questionnaires received online shopping points as incentive. A population-based cross-sectional survey targeting regional working adults was conducted through the Internet in August 2014. We requested registered participants of MARSH Co., Ltd., an Internet research company in Japan, which collected answers from more than 720,000 registered customers, to cooperate in this study. The age eligibility was 20 to 59 years of age. In addition, we excluded students, the unemployed, clergy, and instructors from the study. As a result, we administered the questionnaire to 2,404 adults in Japan, from which 1,134 remained as final participants. The socio-demographic characteristics of the participants are summarized in Table 1.

**Table 1:** Participant characteristics.

	Study Participants(N = 1,134)	
	n	%
**Age**		
20–29	90	7.9
30–39	308	27.2
40–49	419	37.0
50–59	315	27.8
60–69	2	0.2
**Sex**		
Male	558	49.2
Female	576	50.8
**Education**		
Elementary or Junior High	14	1.2
High or Vocational	342	30.2
Junior College or Higher Vocational	158	13.9
College	562	49.6
Graduate School or higher	58	5.1
**Income(Japanese yen)**		
less than 2,000 thousand	67	5.9
2,000–5,000 thousand	381	33.6
5,000–7,000 thousand	238	21.0
7,000–10,000 thousand	228	20.1
10,000–15,000 thousand	102	9.0
more than 15,000 thousand	36	3.2
unknown	82	7.2

Based on ethical guidelines for epidemiological research in Japan [8], an ethical review was not required for this study for the following reasons: 1) based on the Act on the Protection of Personal Information in Japan, the authors provided the participants with the privacy policy, and the participants agreed on the use of their answers for analysis under anonymity; 2) The analysis involved use of an anonymized publicly available dataset provided by Campus for H., Inc., which was the same dataset that was collected from the questionnaire, and does not fall into the definition of research involving”human subjects” according to the Japanese guidelines. Therefore we did not consult an ethical committee for ethical approval.

### Measurements

#### Grit

The Japanese Grit Scale was developed and its reliability and construct validity were tested by Yoshitsu and Nishikawa [9]. Based on the Grit Scale developed by Duckworth and Quinn [10], the Japanese Grit Scale comprises 12 items using a 5-point scale (1 = not like me at all, 5 = very much like me). Six items describe the perseverance of effort for long-term goals, and the six other items describe consistency of interests (as opposed to frequently changing goals) over time. The observed internal reliability of the Japanese Grit Scale had a Cronbach’s α of .69 for perseverance of effort and .78 for consistency of interest for the previous study by Yoshitsu and Nishikawa[9]. However, Yoshitsu and Nishikawa showed in an exploratory factor analysis that questions 3 and 8 (“My interests change from year to year” and “I have difficulty maintaining my focus on projects that take more than a few months to complete”, respectively), after translation into Japanese from the original Grit Scale, did not load onto the consistency of interest factor [9]. Therefore, for the current study, we excluded the data for questions 3 and 8 of the Japanese Grit Scale and scored consistency of interest based on the remaining four questions. Chronbach’s αs for the current study sample were, .89 for perseverance of effort with 6 questions, .76 for consistency of interests with 4 questions, and .87 for grit as a whole with 10 questions.

#### Orientations to happiness

The Orientations to Happiness measure developed by Peterson *et al*. [11] identifies the extent to which respondents feel happiness pursuing pleasure, meaning, and engagement in life. We used the scale translated into Japanese by Kumano [6]. Accordingly, the scale comprises three subscales, each including six items that are rated on a 5-point scale (1 = not like me at all, 5 = very much like me): engagement (e.g., “In choosing what to do, I always take into account whether I can lose myself in it.”), meaning (e.g., “In choosing what to do, I always take into account whether it will benefit other people.”), and pleasure (e.g., “In choosing what to do, I always take into account whether it will feel pleasurable.”). The internal consistency values reported in Peterson *et al*. [11] for the pleasure, engagement, and meaning subscales were .80, .72, and .81, respectively. The Chronbach’s αs for the Japanese version tested by Kumano were .67, .60, .69, for the pleasure, engagement, and meaning respectively [6]. We tested Chronbach’s αs for the current study sample, and had .81, .87, and .75 for the pleasure, engagement, and meaning subscales respectively.

#### Big Five personality traits

The Big Five Scale of personality trait adjectives developed by Wada [12], which is commonly used in Japan, examines a respondent’s personality based on the five-factor model. We used a short form of the Japanese Big-Five Scale developed by Namikawa *et al*. [13], which comprises 29 items rated on a 5-point scale (1 = not like me at all, 5 = very much like me). Five items are for examining Extraversion (α = .86), 7 items for Conscientiousness (α = .78), 5 items for Neuroticism (α = .82), 6 items for Openness to Experience (α = .76), and 6 items for Agreeableness (α = .78). The construct validity of the short form was confirmed to have high correlations (*r* ≥ .93 for all items) with the original Big Five Scale, which comprises 60 items. We also tested Chronbach’s αs for the current study sample, and had .56 for Extraversion, .52 for Conscientiousness, .86 for Neuroticism, .85 for Openness to Experience and .37 for Agreeableness.

#### Self-Control

The Self-Control Scale developed by Tangney *et al*. [14] examines a respondent’s capacity to exert self-control. According to the authors, self-control comprises five main factors: Self-Discipline, Deliberate/Nonimpulsive Action, Healthy Habits, Work Ethic, and Reliability. The scale comprises 36 items using a 5-point scale (1 = not like me at all, 5 = very much like me) and examines the overall self-control level. We used the Japanese Self-Control Scale translated by Hiruma [15]. The reliability of the original scale [14] was confirmed with α ≥ .85. We also tested a Chronbach’s α for the Japanese scale for the current study sample, and had .83.

#### Work Engagement

The UWES was developed by Schaufeli *et al*. [16] as a self-report questionnaire that asks how often a respondent currently experiences positive emotions at work and that assesses three dimensions of work engagement—vigor, dedication, and absorption—using a 7-point scale (0 = never, 6 = always). A 9-item short version of the scale (UWES-9) was developed by Schaufeli *et al*. [17] and has sufficient internal reliability and construct validity. The Japanese version of the original UWES was developed by Shimazu *et al*. [18] and was confirmed to have sufficiently high internal consistency and test-retest reliability; the 9-item short version of the Japanese scale (Japanese UWES-9) has been used more recently but has not been tested yet for reliability or construct validity [19]. We employed the Japanese UWES-9 for the current study, and tested a Chronbach’s α for the Japanese UWES-9 for the current study sample, and had .96.

### Analysis

We used the statistic software package R 3.1.1 for the analysis of multiple regression models. For the question 1, we analyzed associations between three orientations to happiness as explanatory variables and grit as an outcome variable (Model 1). Model 2 added age, sex, income, and education, and Model 3 added Big Five traits and self-control as explanatory variables. For the question 2, we analyzed the association between grit as an explanatory variable and work engagement as an outcome variable. After the crude analysis (Model 1), Model 2 added age, sex, income, and education, while Model 3 added orientations to happiness, Big Five traits, and self-control as explanatory variables.

## Results

### Question 1: Grit and Orientations to Happiness

The results of the multiple regression analysis were presented in Table 2. Each of the three orientations to happiness was significantly associated with grit (*r* = .26, *p* < .001, Model 1), and these results were consistent even after controlling for age, sex, income, education, Big Five personality traits, and self-control (Model 3). An orientation to meaning was more strongly associated with grit (β = 0.029 for Model 3) than was an orientation to engagement (β = 0.014 for Model 3), while an orientation to pleasure was inversely associated (β = -0.020 for Model 3). Among all the factors in Model 3, sex had the strongest association with grit (β = 0.067, *p* < .01), which suggests that women are more likely to be gritty. Conscientiousness (β = 0.020, *p* < .001), Extraversion (β = 0.008, *p* < .01), and Openness to Experience (β = 0.013, *p* < .001) within the Big Five personality traits and self-control (β = 0.010, *p* < .001) were also positively correlated with grit. We further conducted a secondary analysis to test for interactions between orientations towards happiness and sex in Model 4. As a result, sex did not show a statistically significant interaction effect with any category of orientation towards happiness.

**Table 2:** Associations of grit with orientations to happiness, demographics, personality traits, and self-control.

Coefficients	Grit									
	Model 1		Model 2		Model 3		Model 4			
	β	p	β	p	β	p	β	p	Adj. R2	ΔR2
**1**									0.26	
(Intercept)	2.117***	<0.001	1.452***	<0.001	-0.272	0.144	-0.188	0.445		
Happiness_Engagement	0.019***	0.0007	0.019***	0.0006	0.014**	0.002	0.019	0.174		
Happiness_Meaning	0.052***	<0.001	0.052***	<0.001	0.029***	<0.001	0.021	0.052		
Happiness_Pleasure	-0.031***	<0.001	-0.030***	<0.001	-0.020***	<0.001	-0.025	0.103		
**2**									0.29	0.03
Age			0.008***	<0.001	0.002	0.208	0.002	0.205		
Sex			0.267***	<0.001	0.067**	0.005	0.004	0.973		
Income			0.010	0.2676	-0.002	0.793	-0.001	0.842		
Education			0.018	0.2040	0.013	0.274	0.012	0.293		
**3**									0.52	0.23
Big 5_Conscientiousness					0.020***	<0.001	0.02***	<0.001		
Big 5_Extraversion					0.008**	0.002	0.008**	0.002		
Big 5_Neuroticism					0.002	0.388	0.002	0.367		
Big 5_Openness					0.013***	<0.001	0.013***	<0.001		
Big 5_Agreeableness					0.001	0.835	0.001	0.838		
Self Control					0.010***	<0.001	0.01	<0.001		
**4**										
Happiness_Engagement*Sex							-0.004	0.687		
Happiness_Meaning*Sex							0.005	0.487		
Happiness_Pleasure*Sex							0.003	0.725		

**p < .01.

***p < .001.

Table 3 shows another secondary analysis for the subscales of grit, perseverance of effort and consistency of interest. With regard to orientations to happiness, both engagement and meaning were more strongly and positively associated with perseverance (β = 0.037, *p* < .001 and β = 0.049, *p* < .001, respectively) and inversely associated with consistency (β = -0.021, *p* < .01 and—β = 0.002, *p* = 0.658, respectively). Conscientiousness, Extraversion, and Openness to Experience were more strongly associated with perseverance (β = 0.0247, 0.0116, and 0.0253, respectively, all *p* < .001) than with consistency. Self-control was positively associated with both perseverance (β = 0.007, *p* < .001) and consistency (β = 0.015, *p* < .001).

**Table 3:** Associations of grit subscales with orientations to happiness, self-control, personality traits, and demographics.

Coefficients	Grit			
	Perseverance of Effort		Consistency of Interest	
	β	p	β	p
(Intercept)	-1.753***	<0.001	1.949***	<0.001
Happiness_Engagement	0.037***	<0.001	-0.021**	0.001
Happiness_Meaning	0.049***	<0.001	-0.002	0.658
Happiness_Pleasure	-0.011	0.084	-0.033***	<0.001
Age	0.002	0.295	0.001	0.451
Sex	0.086**	0.006	0.038	0.272
Income	-0.013	0.183	0.014	0.173
Education	0.039**	0.009	-0.027	0.099
Big 5_Conscientiousness	0.025***	<0.001	0.012**	0.001
Big 5_Extraversion	0.011***	<0.001	0.002	0.505
Big 5_Neuroticism	0.010**	0.002	-0.009**	0.006
Big 5_Openness	0.026***	<0.001	-0.006	0.123
Big 5_Agreeableness	0.001	0.785	0.000	0.990
Self Control	0.007***	<0.001	0.015***	<0.001

**p < .01.

***p < .001.

### Question 2: Work Engagement and Grit

The results of the multiple regression analysis were presented in Table 4. Grit had a significant positive association with work engagement (*r* = .26, *p* < .001), and this result was consistent even after controlling for other variables in Models 2 and 3. Age (β = 0.010, *p* < .01 in Model 3) and education (β = 0.055, *p* < .05 in Model 3) were positively associated with work engagement. With regard to orientations to happiness, both engagement and meaning were positively associated with work engagement (β = 0.060 and 0.057, respectively, both *p* < .001), while pleasure was inversely associated with work engagement (β = -0.051, *p* < .001). Furthermore, the Big Five traits of Extraversion (β = 0.015, *p* < .05) and Openness to Experience (β = 0.019, *p* < .01) were positively associated with work engagement

**Table 4:** Associations between Work Engagement and Grit.

Coefficients	Work Engagement									
	Crude Model 1		Model 2		Model 3		Model 4			
	β	p	β	p	β	p	β	p	Adj. R2	ΔR2
**1**									0.26	
(Intercept)	-0.741***	<0.001	-1.292***	<0.001	-1.946***	<0.001	-2.10**	0.001		
Grit	1.095***	<0.001	1.06***	<0.001	0.472***	<0.001	0.52**	0.002		
**2**									0.28	0.02
Age			0.01**	0.003	0.010**	0.001	0.01**	0.001		
Sex			-0.091	0.130	-0.009	0.876	0.09	0.773		
Income			0.028	0.142	-0.001	0.953	-0.001	0.950		
Education			0.084**	0.005	0.055*	0.041	0.055*	0.040		
**3**									0.42	0.14
Happiness_Engagement					0.060***	<0.001	0.060***	<0.001		
Happiness_Meaning					0.057***	<0.001	0.057***	<0.001		
Happiness_Pleasure					-0.051***	<0.001	-0.050***	<0.001		
Big 5_Conscientiousness					0.001	0.900	0.001	0.917		
Big 5_Extraversion					0.015*	0.013	0.015*	0.013		
Big 5_Neuroticism					-0.009	0.105	-0.009	0.110		
Big 5_Openness					0.019**	0.003	0.019**	0.004		
Big 5_Agreeableness					0.005	0.377	0.006	0.374		
Self Control					0.001	0.743	0.001	0.732		
**4**										
Grit*Sex							-0.031	0.747		

*p < .05.

**p < .01.

***p < .001.

In Model 4 we conducted the secondary analysis to test for an interaction between grit and sex. As a result, sex did not show a statistically significant interaction effect with grit.

## Discussion

The aims of the current study were (1) to replicate the previous study by Von Culin *et al*. [5] by testing associations between grit and orientations to happiness, and (2) to test the association between grit and work performance. To our knowledge, this is the first study that examined the relationship between work performance and grit. Despite the limitation of using a cross-sectional design, the current results will have useful implications for future studies in the field.

### Question 1: Grit and Orientations to Happiness

The most important finding was that Japanese people are more likely to be grittier if they seek happiness through meaning rather than engagement. Consistent with Von Culin *et al*. [5], orientations to engagement and meaning had positive associations with grit, while an orientation to pleasure had a negative association. However, while their U.S. study showed the strongest association between engagement and grit, our study showed the strongest association between meaning and grit. This difference may be explained by Japanese people’s mentality, which tends to value contribution to and consolidation of the organization or society to which they belong more than individual engagement, whereas the opposite appears true in the U.S. (e.g., [20], [21])

The second important finding was that the Big Five trait of Openness to Experience had a positive association with grit. When we divided grit into its components, perseverance of effort and consistency of interest, Openness to Experience showed a stronger association with perseverance of effort than with consistency of interest. As Barrick and Mount [3] explained, Openness to Experience involves a facet of curiosity, and according to Nye *et al*. [22], curiosity is related to perseverant academic and occupational performance. This factor can explain the strong association between Openness to Experience and grit, especially in the domain of perseverance of effort. According to Duckworth *et al*.’s [4,5] hypothesis, grit, especially the subcomponent consistency of interest, may share similar elements with Conscientiousness and self-control. Consistent with this hypothesis, our results showed positive associations between grit and Conscientiousness and self-control.

Inconsistent with Von Culin *et al*. [5], not only an orientation towards pleasure but also orientations towards engagement and meaning showed negative associations with consistency of interest, although the association of meaning was not statistically significant. There are two possible explanations for this result. First, it might be caused by the incomplete translation from the original Grit Scale to the Japanese version. Second, since objectives and missions frequently change in the workplace, the gritty working adults may not necessarily be consistent in their interests but still perseverant in their work.

### Question 2: Work Engagement and Grit

For the second research question, we tested the association between grit and work engagement. First, consistent with our hypothesis, grittier individuals were more likely to engage with their work than were less gritty individuals, independent of age, sex, income, education level, orientations to happiness, Big Five traits, and self-control. Second, with regards to other personality traits as controlling factors, several traits showed significant results. People who seek happiness through engagement and meaning are similarly likely to feel engaged with their work. This may be explained in the context of the workplace, where people are frequently exposed to opportunities to feel or think about the social meaning of their work, and if they find importance in their work, they may feel happier and more engaged to performing it. Therefore, not only personal engagement but also social meaning affects their work engagement. On the other hand, those who seek happiness through pleasure were less likely to be engaged with their work.

The third significant finding was that among the Big Five traits, Openness to Experience and Extraversion had strong positive associations with the UWES, while the other three traits—Conscientiousness, Agreeableness, and Neuroticism—had weaker associations. Previously, Barrick and Mount [3] showed that Conscientiousness was an important performance predictor for all occupational groups, while Extraversion was a valid predictor for occupations involving social interaction such as managers and salespersons. The results of the current study can be explained as follows: First, in today’s society, industrial trends change more frequently, and stakeholders are more widely and closely connected than ever. Therefore, individuals who have a keen interest for new information and experience and who are socially extroverted and excel at networking adapt more easily and become more strongly engaged with such a fast-moving working environment than those who are mostly conscientious. Second, since the UWES examines not only individual performance but also social interaction and work flexibility in the complex organizational environment, Extraversion and Openness to Experience may be more strongly associated with the outcome than Conscientiousness.

## Limitations

This study has several limitations. First, fewer participants were in their 20s and 60s compared with participants between the ages of 30 and 50 years. Therefore, it is possible that the current results do not properly reflect the characteristics of the youngest and oldest working groups. Second, as we mentioned above, the Japanese Grit Scale, specifically the subscale of consistency of interest, may have been imperfectly translated from the original version, which may have distorted the analysis. Third, it is possible that our significant results were influenced by the large sample size, because partial regression coefficients in our analysis were small. Forth, since the study was a cross-sectional design, we can only speculate about sequential changes between explanatory variables and outcomes in the same person. To monitor differences in work performance between grittier and less gritty individuals, prospective cohort studies will be required. It is further possible that respondents in our study differed with respect to unobservable factors that influenced both explanatory and outcome variables. For example, on the basis of Holland’s [23,24] theoretical predictions, Nye *et al*. [22] showed that correlations between congruence indices, which quantify the degree of similarity between individuals and their occupations, and performance were stronger than those between interest scores and performance. Further analyses that include occupational and environmental classifications need to be conducted to uncover conditions in which less gritty people can engage more strongly with their work.

## Conclusion

In this study, we examined the relationship between grit and orientations towards happiness for Japanese population, replicating the previous study by Von Culin *et al*. [5] The result showed that Japanese people are likely to be grittiest if they seek happiness through meaning, while U.S. people to be grittiest if they seek happiness through engagement. Also, we conducted the first study that examined the relationship between work performance and grit for Japanese population. The result showed that gritty people are likely to engage positively in their work. These findings will have useful implications for future studies in the field.

## Supporting Information

S1 Dataset Grit and Work Engagement. (XLSX)
